# Supplementary material for: Single-cell RNA sequencing shows the immunosuppressive landscape and tumor heterogeneity of HBV-associated hepatocellular carcinoma
Source: Nat Commun. 2021 Jun 17;12:3684. doi: 10.1038/s41467-021-24010-1 (PMC8211687; doi:10.1038/s41467-021-24010-1)
Supplement: Supplementary file 1 — Supplementary Information [file 41467_2021_24010_MOESM1_ESM.pdf]

Single-cell RNA sequencing shows the immunosuppressive landscape and tumor heterogeneity of HBV-associated hepatocellular carcinoma

Daniel Wai-Hung Ho[1,2,*], Yu-Man Tsui[1,2], Lo-Kong Chan[1,2], Karen Man-Fong Sze[1,2], Xin Zhang[1,2], Jacinth Wing-Sum Cheu[1], Yung-Tuen Chiu[1,2], Joyce Man-Fong Lee[1,2], Albert Chi-Yan Chan[2,3], Elaine Tin-Yan Cheung[4], Derek Tsz-Wai Yau[4], Nam-Hung Chia[5], Irene Lai-Oi Lo[5], Pak-Chung Sham[6], Tan-To Cheung[2,3], Carmen Chak-Lui Wong[1,2], Irene Oi-Lin Ng[1,2,*]

[1]Department of Pathology, The University of Hong Kong

[2]State Key Laboratory of Liver Research, The University of Hong Kong

[3]Department of Surgery, The University of Hong Kong, Hong Kong

[4]Department of Pathology, Queen Elizabeth Hospital, Hong Kong

[5]Department of Surgery, Queen Elizabeth Hospital, Hong Kong

[6]Department of Psychiatry, The University of Hong Kong, Hong Kong

*To whom correspondence should be addressed:

Daniel WH Ho, email: dwhho@hku.hk, Lab 704, 7th Floor, Laboratory Block, HKU Medical Center, 21 Sassoon Road, Pokfulam, Hong Kong or Irene Oi-Lin Ng, Room 7-13, Block T, Queen Mary Hospital, Pokfulam, Hong Kong. E-mail: iolng@hku.hk.

Supplementary Information

Supplementary Figure 1. QC checking of scRNA-seq data.

Supplementary Figure 2. Batch effect checking by PCA.

Supplementary Figure 3. Highly variable gene selection and PC heatmap.

Supplementary Figure 4. Cell type marker, tumor marker and LCSC marker expressions in single cells.

Supplementary Figure 5. Identification of malignant and non-malignant cells in HCC tumors.

Supplementary Figure 6. Heatmap for top 20 upregulated genes for the 34 cell clusters identified by unsupervised clustering.

Supplementary Figure 7. Gene expression of cell type and immunosuppressive markers in different cell clusters.

Supplementary Figure 8. Inverse correlation between the proportions of T cells and TAMs in scRNA-seq and deconvoluted bulk-cell RNA-seq datasets (in-house, TCGA LIHC and the subset of TCGA HBV+ LIHC).

Supplementary Figure 9. IHC staining of LAIR1 and CD163.

Supplementary Figure 10. Knockdown of LAIR1 in THP-1 cells reduced macrophage proliferation and upregulated CD8 T cell activation.

Supplementary Figure 11. Gene expression pattern of complementary co-inhibitory immune checkpoint molecules.

Supplementary Figure 12. NECTIN2 is the most prominently expressed in PVR family that interacts with TIGIT.

Supplementary figure 13. IHC staining of TIGIT and NECTIN2.

Supplementary Figure 14. Comparison of IHC of NECTIN2 on HCCs and the corresponding non-tumorous livers.

Supplementary Figure 15. Effect of Nectin2 KO in HCC.

Supplementary Figure 16. Cell-cell communication between immune cells and APCs (TAMs and tumor cells) via ligand-receptor interactions.

Supplementary Figure 17. HCC tumor cells stratified according to global transcriptomic profile.

Supplementary Figure 18. LCSC marker expression status of HCC tumor cells.

Supplementary Figure 19. CNV profile and the derived lineage hierarchy of tumor cells.

Supplementary Figure 20. CNV status of HCC tumor cells.

Supplementary Figure 21. Gene expression of RTK families in HCC tumor cells.

Supplementary Figure 22. Gating strategies for tumor-infiltrating lymphocytes analysis in Figure 5 and Supplementary Figure 15.

Supplementary Table 1. Demographic and pathological data of the HCC patients.

Supplementary Table 2. Statistics of the scRNA-seq dataset.

Supplementary Table 3. Gene markers used for cell type identification.

Supplementary Table 4. Cell type composition in different HCC cases.

Supplementary Table 5. Correlation of the LAIR1 and CD163 expression by IHC in human HCC (n=29). Chi-square test (2-sided).

Supplementary Table 6. Correlation of the TIGIT and NECTIN2 expression by IHC in human HCC (n=29). Chi-square test (2-sided).

Supplementary Table 7. Correlation of TIGIT and NECTIN2 expression by IHC in human non-HCC, HBV-associated cirrhotic livers (n=22). Chi-square test (2-sided).

Supplementary Table 8. Distribution of LCSC marker groups in tumor cells of different HCC cases.

Supplementary Table 9. Distribution of CNV groups in tumor cells of different HCC cases.

Supplementary Table 10. Information of the sgRNA and shRNA sequences.

Supplementary Table 11. Information of primary antibodies for flow cytometry, IHC staining and Nectin2 neutralizing antibody.

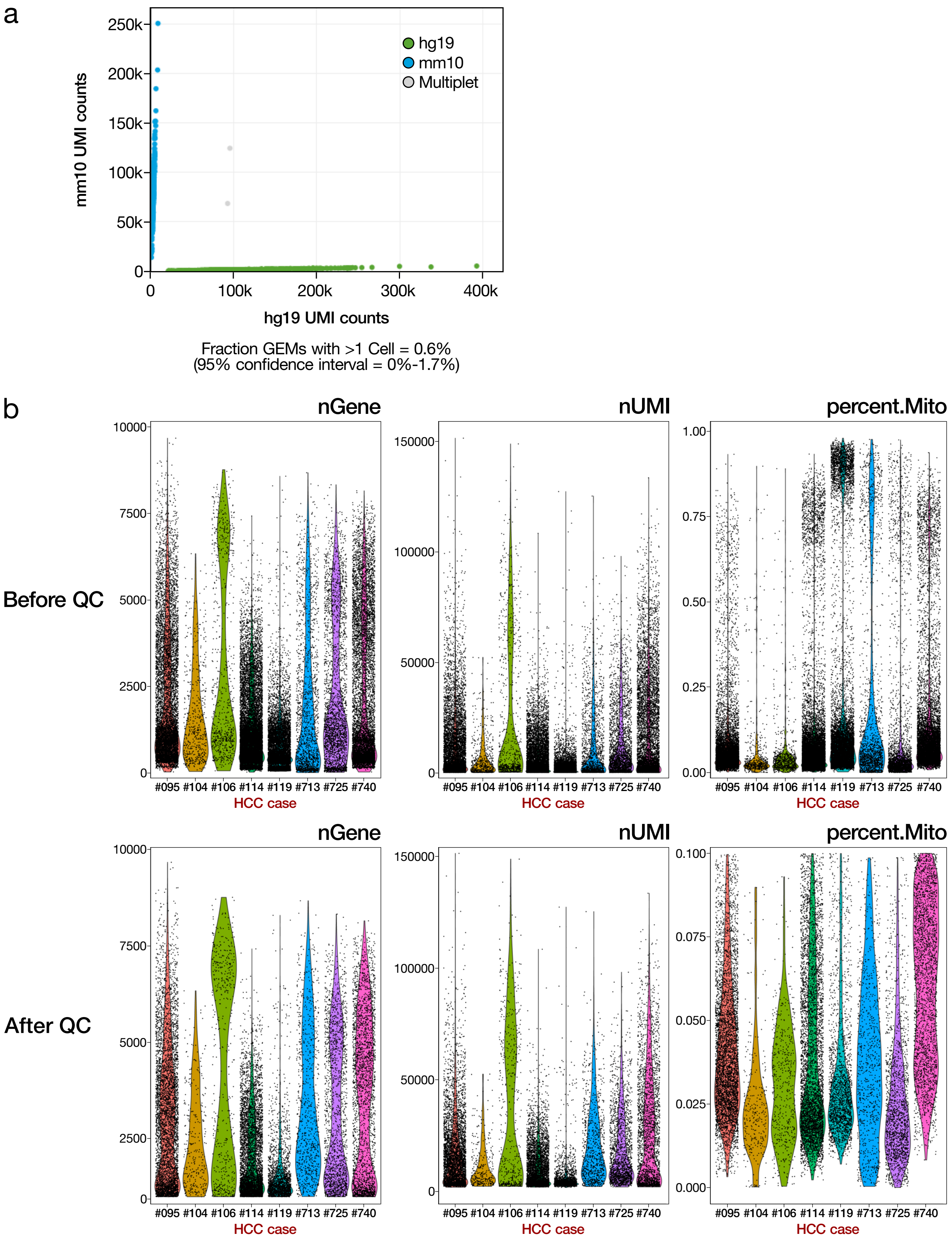

Supplementary Figure 1. QC checking of scRNA-seq data.
(a) Cell multiplet estimation by human and mouse cell mix experiment.
(b) Statistics (nGene, nUMI and percent.mito refer to the number of genes, UMIs, and the percentage of mitochondrial genes respectively) used for quality control filtering. We removed those cases with nGene <1000 and percent.mito >0.1.

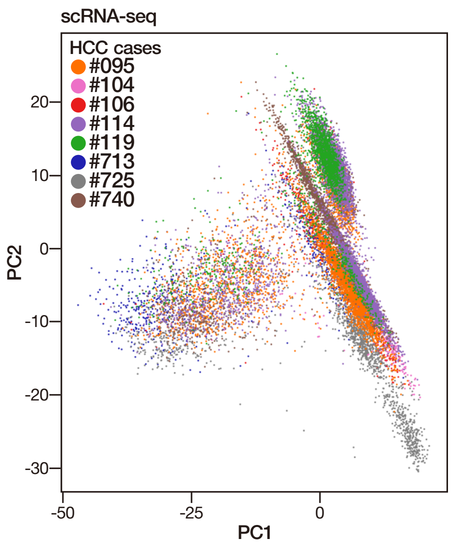

HCC cases	Batch
#095	3
#104	2
#106	2
#114	3
#119	3
#713	1
#725	2
#740	3

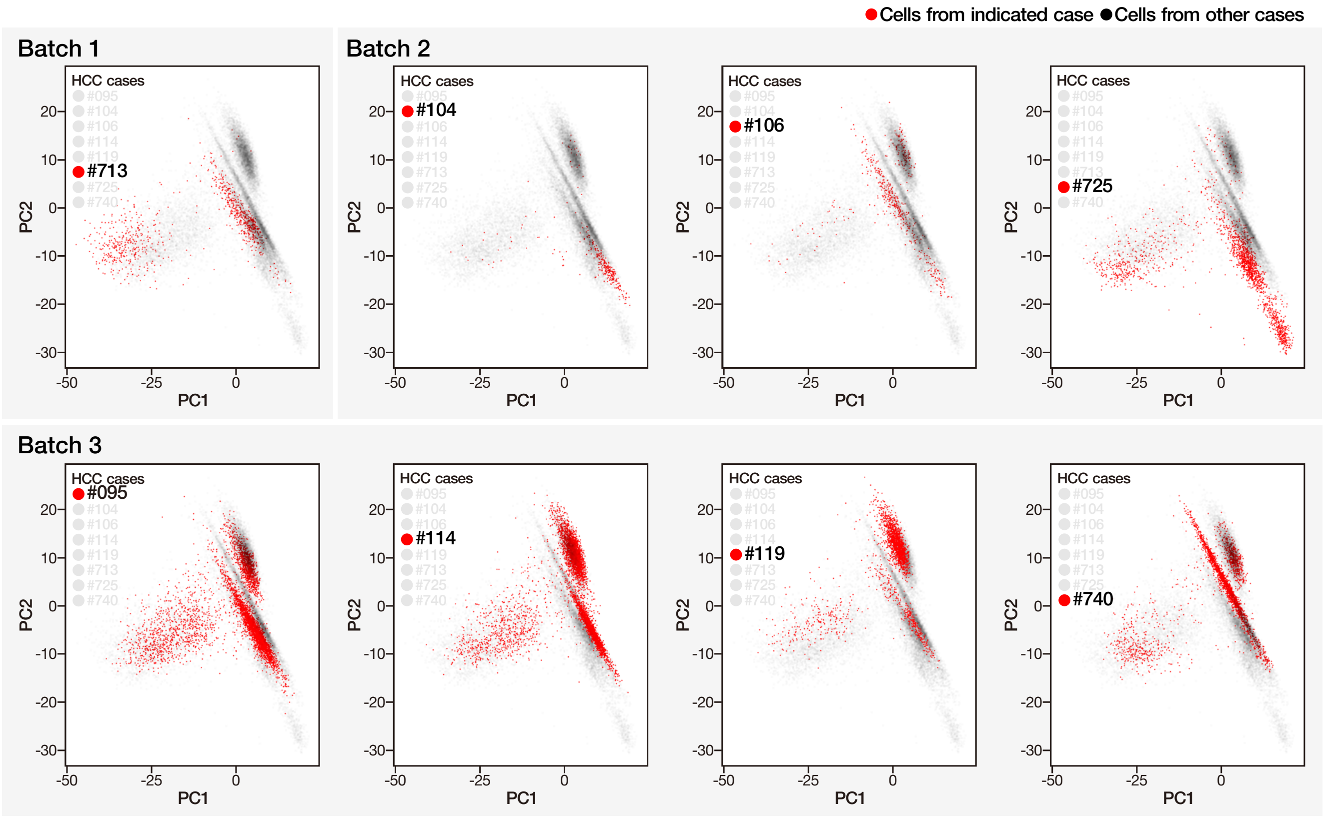

Supplementary Figure 2. Batch effect checking by PCA.

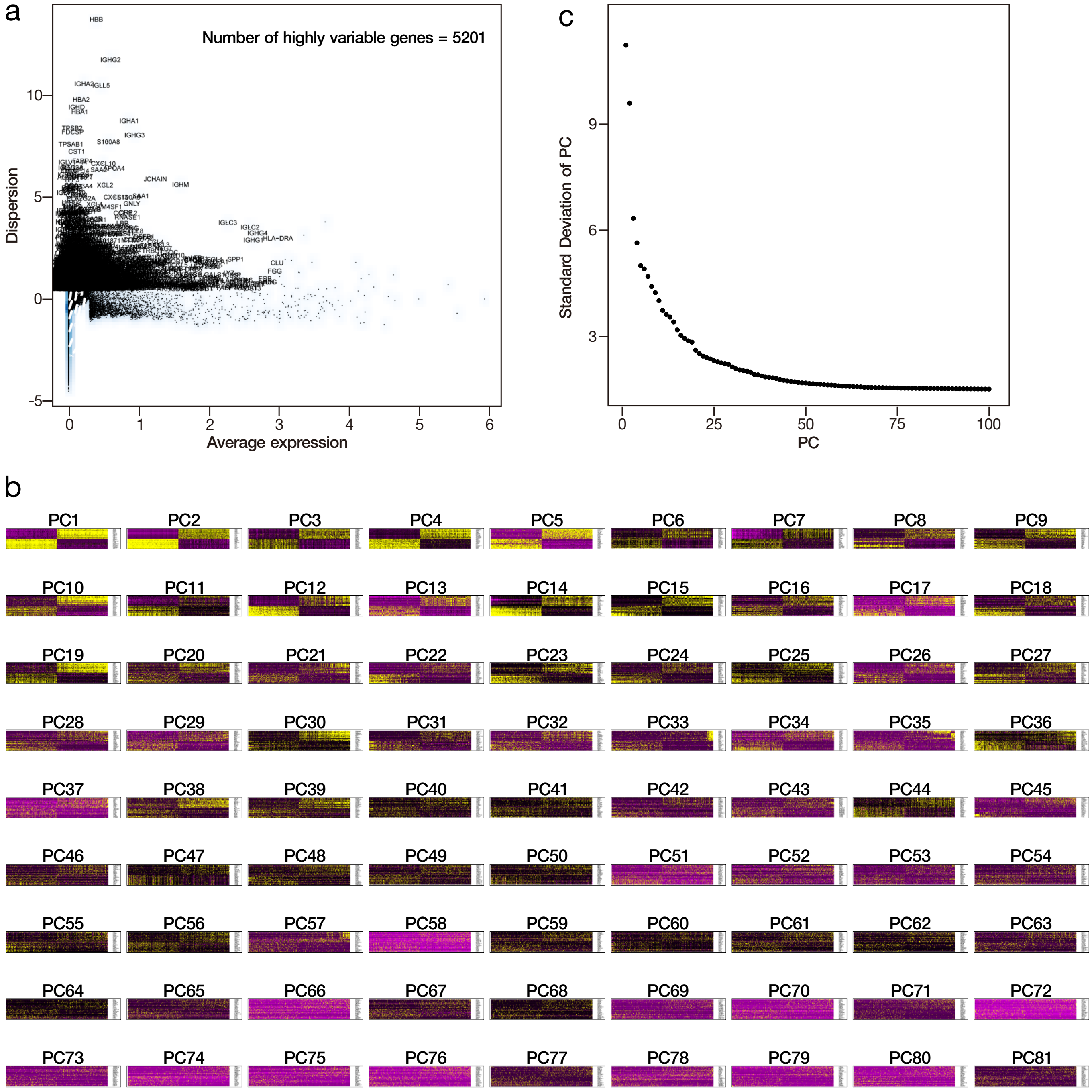

Supplementary Figure 3. Highly variable gene selection and PC heatmap.
(a) Highly variable gene selection by considering average expression and dispersion (mean.function = ExpMean, dispersion.function = LogVMR, x.low.cutoff = 0.0125, x.high.cutoff = 3 and y.cutoff = 0.5).
(b) PC heatmap for the first 81 PCs. c. PC elbow plot for the first 100 PCs.

Cell type	Gene marker
Pan-leukocyte	CD45 (PTPRC)
Monocyte	CD14
Pan-macrophage	CD68
M2 macrophage	CD163
Dendritic cell (DC)	CD11C (ITGAX)
Myeloid-derived suppressor cell (MDSC)	CD11B (ITGAM)<br>CD33
B cell	CD19<br>CD79A
Natural killer cell	CD56 (NCAM1)<br>FCGR3A
Pan-T cell	CD3E
CD4 T cell	CD4<br>IL7R
CD8 T cell	CD8A<br>NKG7
Regulatory T cell	CD4<br>CD25 (IL2RA)<br>FOXP3
Endothelial cell	CD31 (PECAM1)<br>CD34

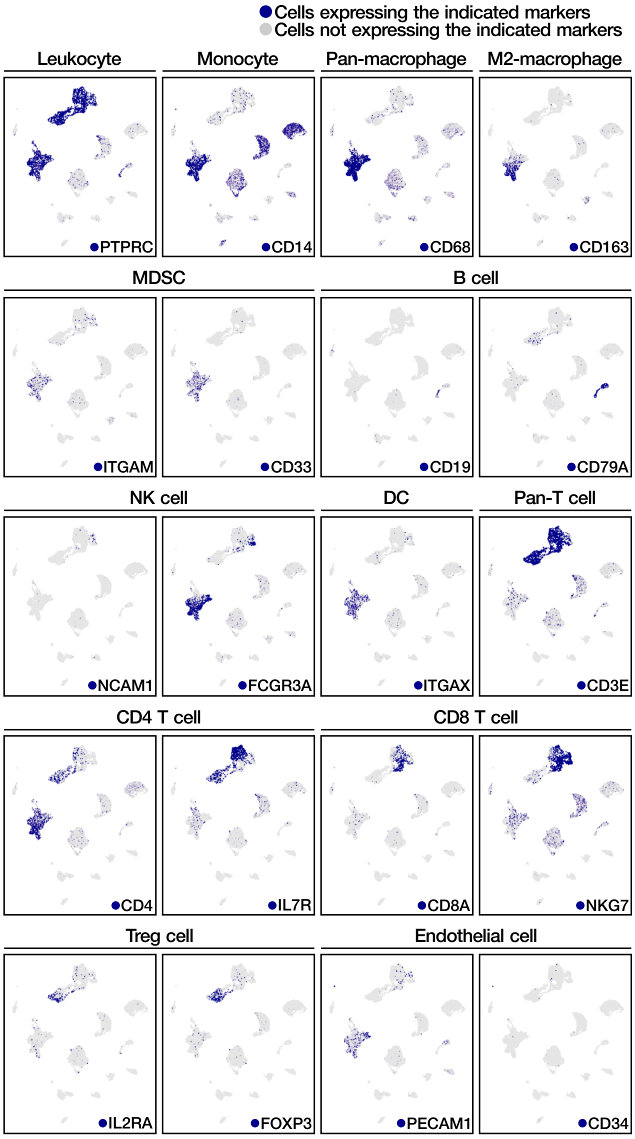

	Gene marker	Protein name
Tumor marker	AFP	AFP
	GPC3	GPC3
LCSC marker	ALDH	ALDH1A1
	EPCAM	EPCAM
	KRT19	KRT19
	CD13	ANPEP
	CD24	CD24
	CD44	CD44
	CD47	CD47
	CD90	THY1
	CD133	PROM1

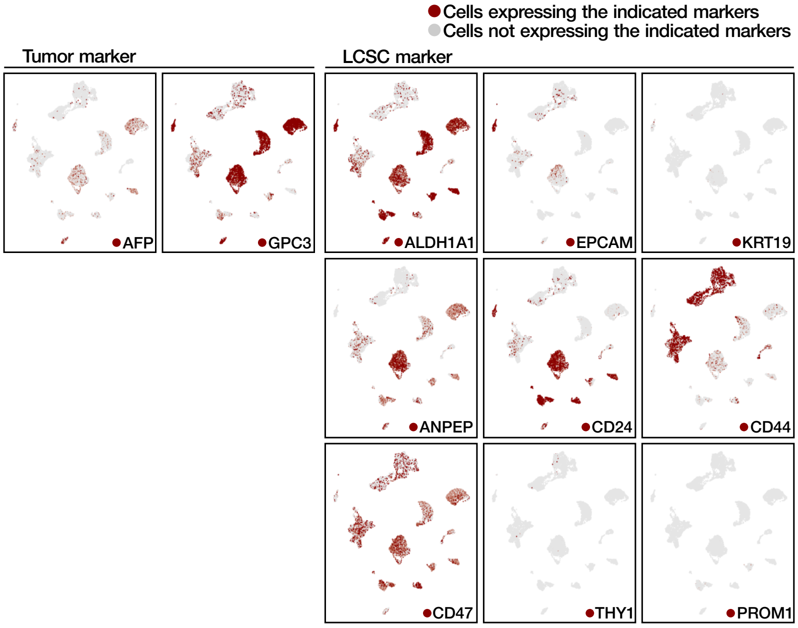

Supplementary Figure 4. Cell type marker, tumor marker and LCSC marker expressions in single cells.

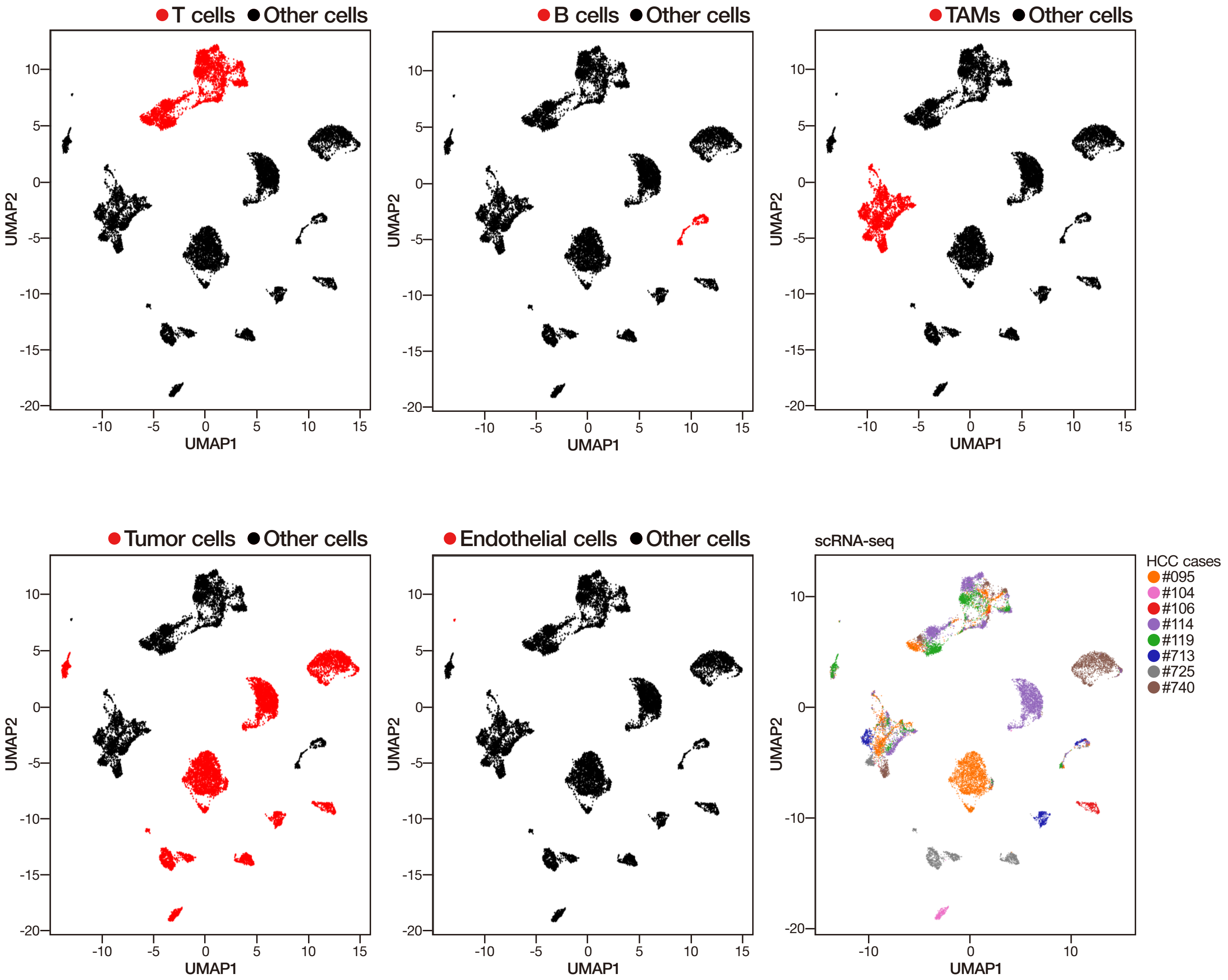

Supplementary Figure 5. Identification of malignant and non-malignant cells in HCC tumors.

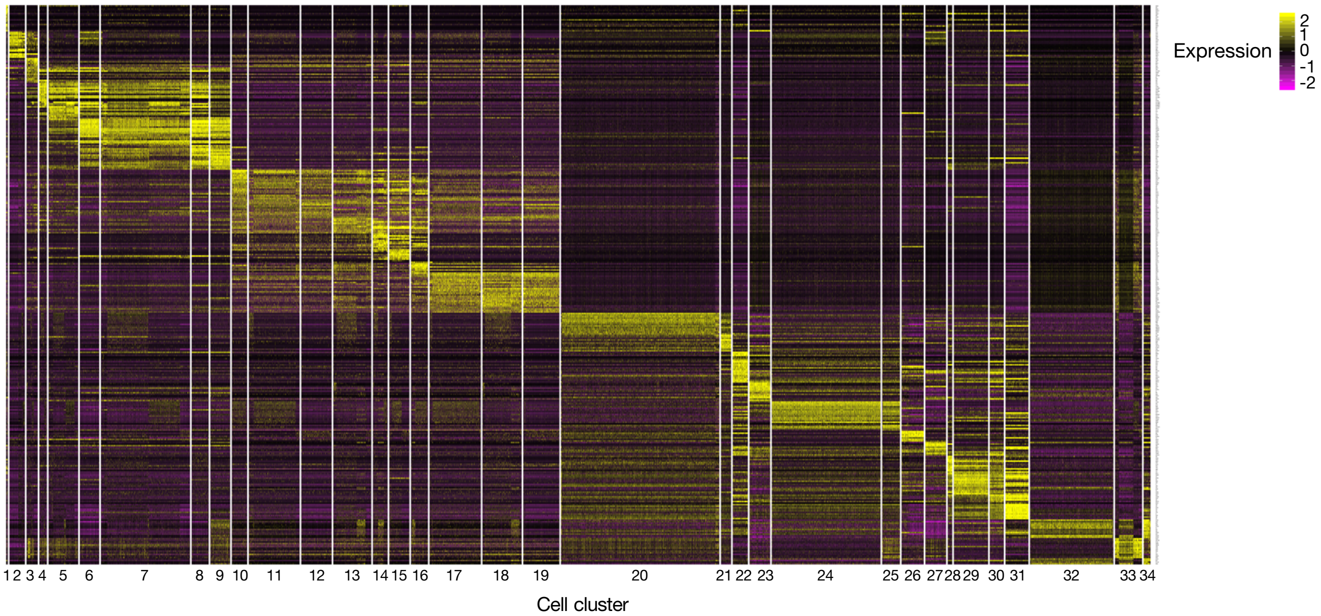

Supplementary Figure 6. Heatmap for top 20 upregulated genes for the 34 cell clusters identified by unsupervised clustering.

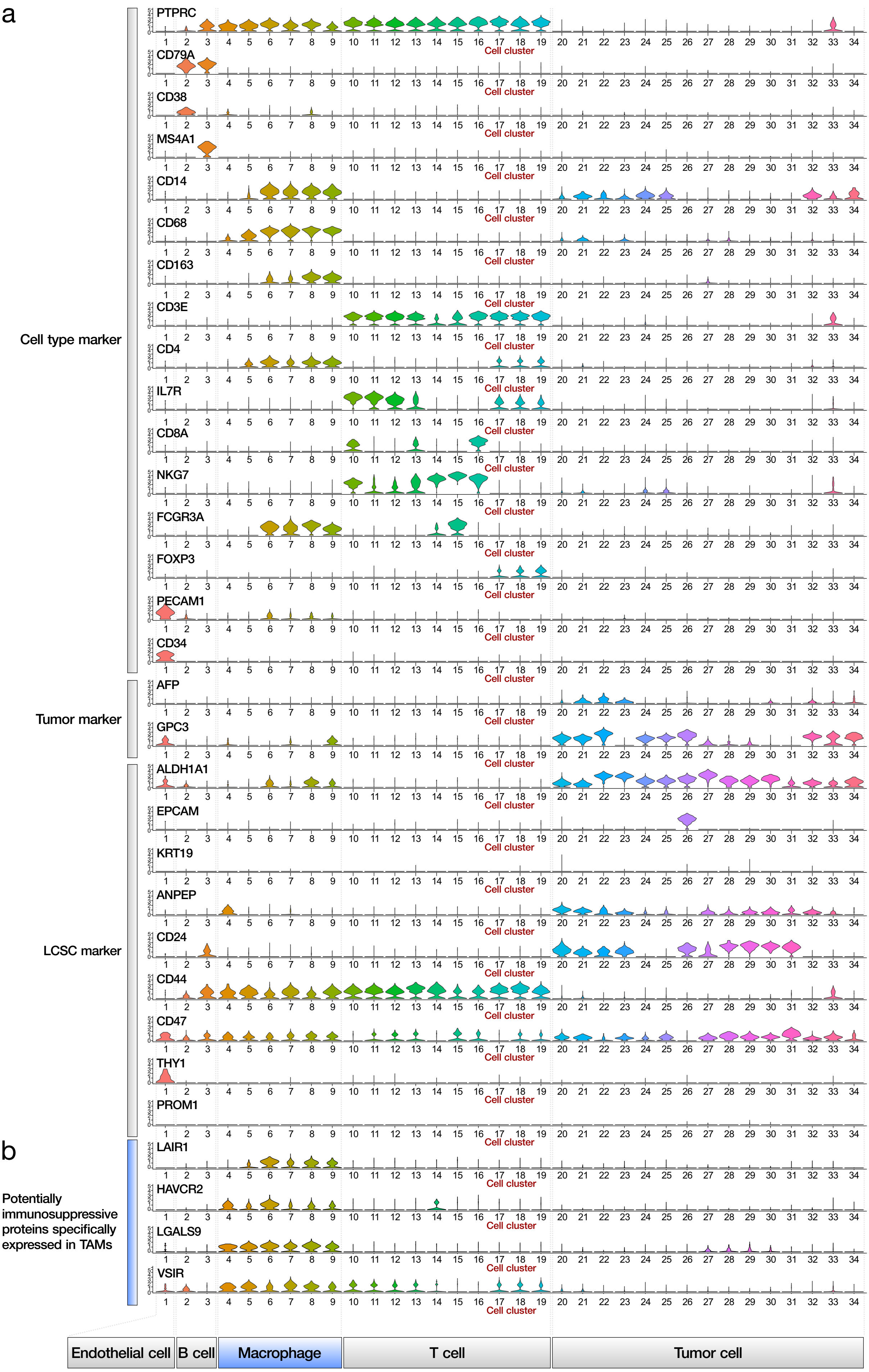

Supplementary Figure 7. Gene expression of cell type and immunosuppressive markers in different cell clusters.
(a) Cell type identification in different cell clusters.
(b) Enriched expression for multiple potentially immunosuppressive proteins in TAMs.

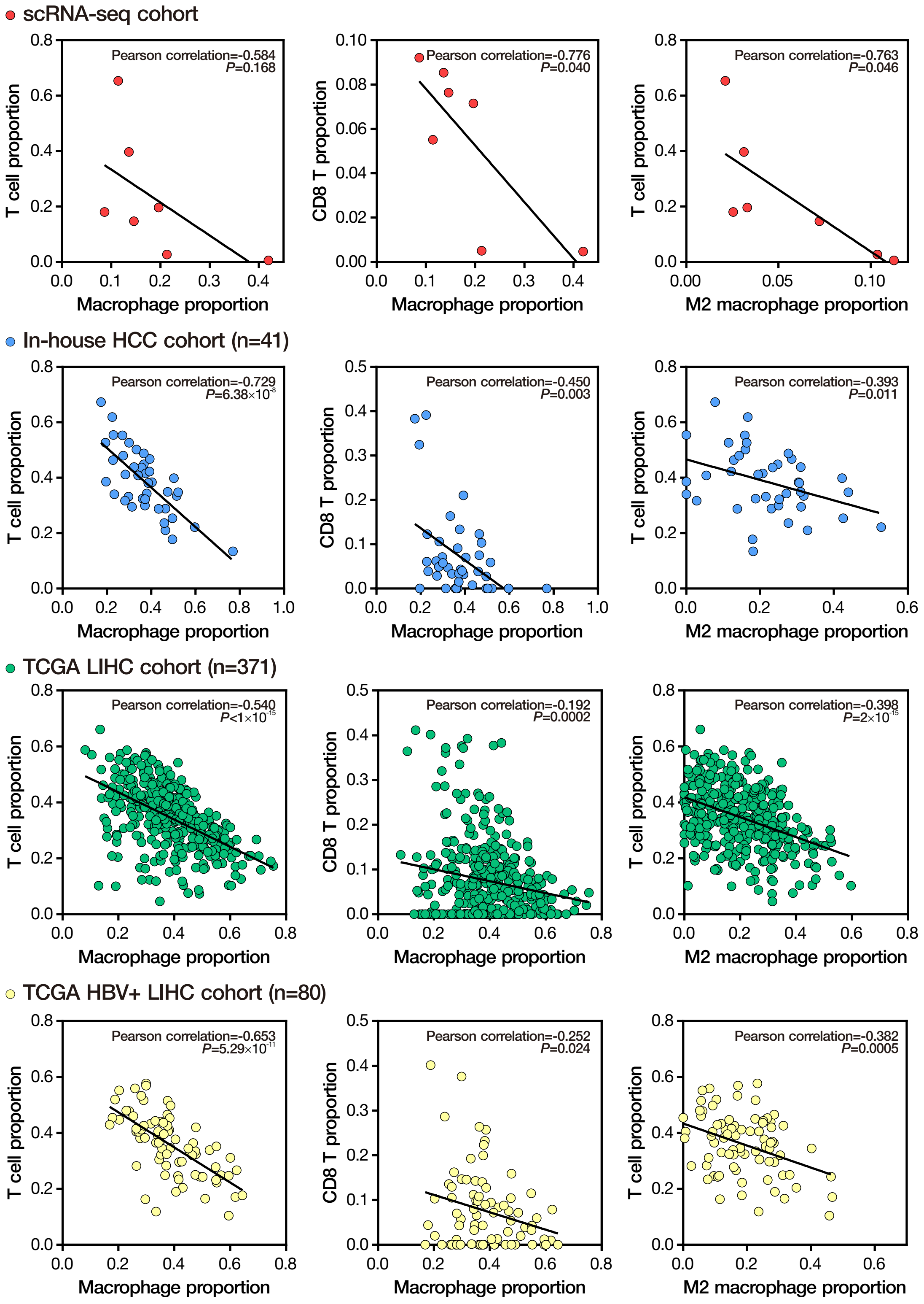

Supplementary Figure 8. Inverse correlation between the proportions of T cells and TAMs in scRNA-seq and deconvoluted bulk-cell RNA-seq datasets (in-house, TCGA LIHC and the subset of TCGA HBV+ LIHC). Pearson correlation (2-sided). Source data are provided as a Source Data file.

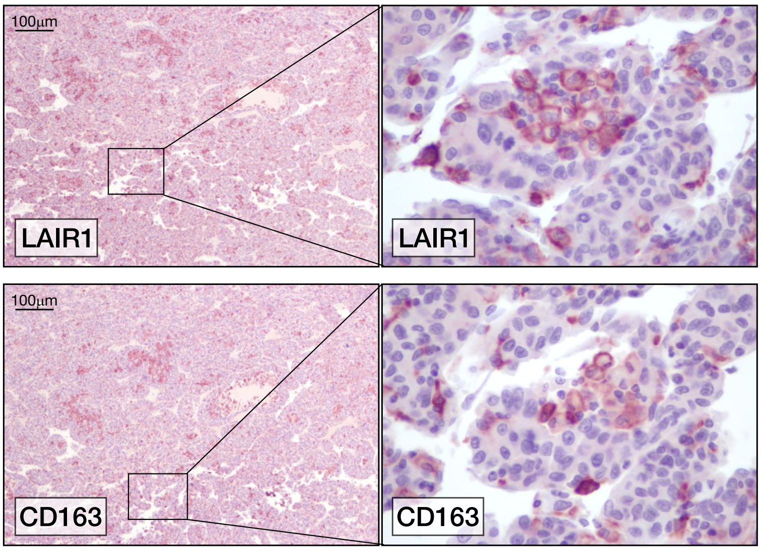

Supplementary Figure 9. IHC staining of LAIR1 and CD163.
A representative case of HCC showing strong IHC positivity of LAIR1 and CD163, with significantly overlapping staining. Scale bar = 100µm. More than 10 fields each under x40 and x100 magnification were examined.

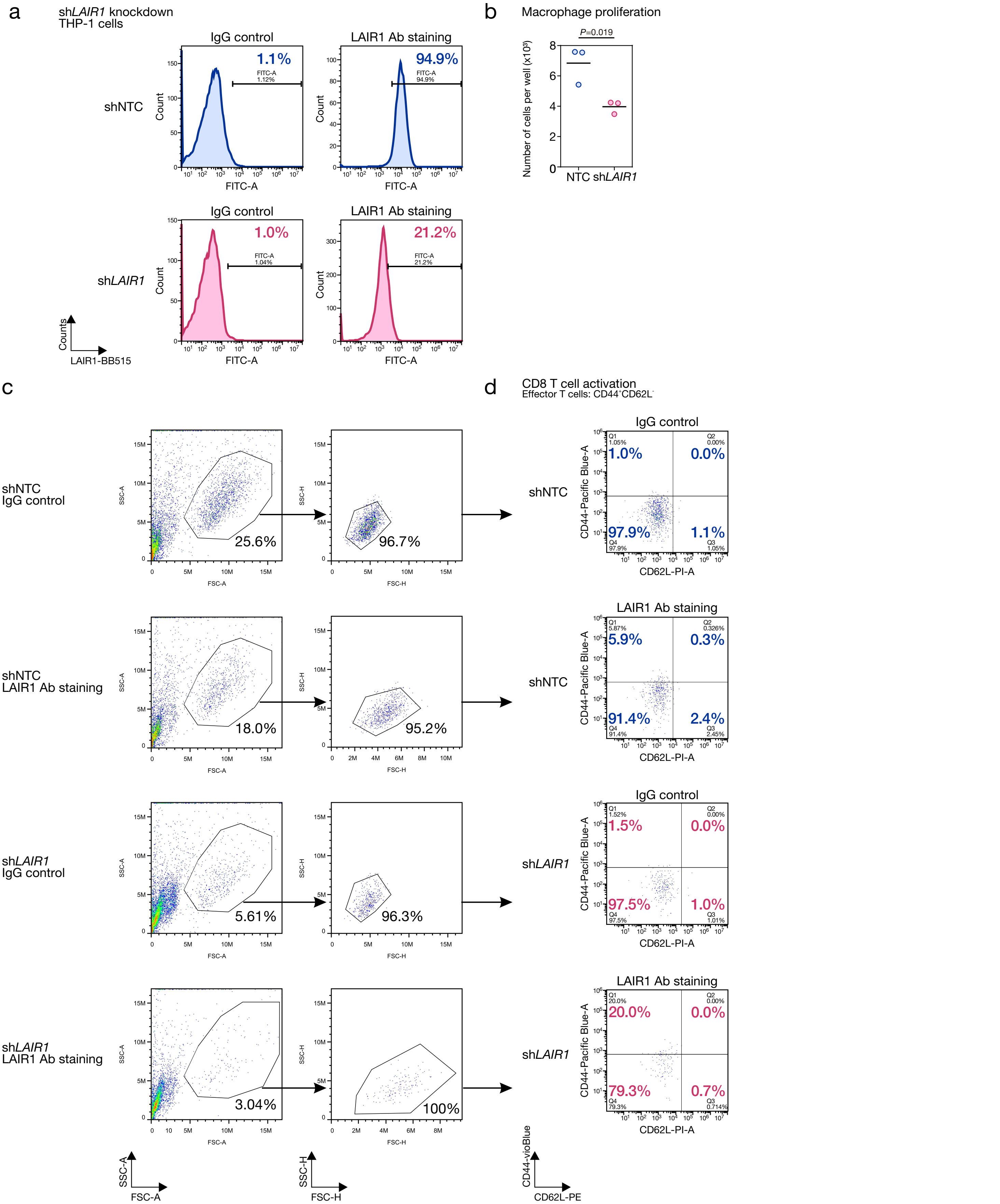

Supplementary Figure 10. Knockdown of LAIR1 in THP-1 cells reduced macrophage proliferation and upregulated CD8 T cell activation.
(a) LAIR1 expression in the control and sh*LAIR1* knockdown THP-1 cells.
(b) Examination of the proliferation rate of the control and sh*LAIR1* macrophages.
(c) The gating strategies used for the flow cytometry analysis for the CD8 T cell activation.
(d) Examination of the degree of CD8 T cell activation (CD44+CD62L- staining) induced by control and sh*LAIR1* knockdown THP-1 cells. Experiment was performed with n=3 biologically independent samples per group. Source data are provided as a Source Data file.

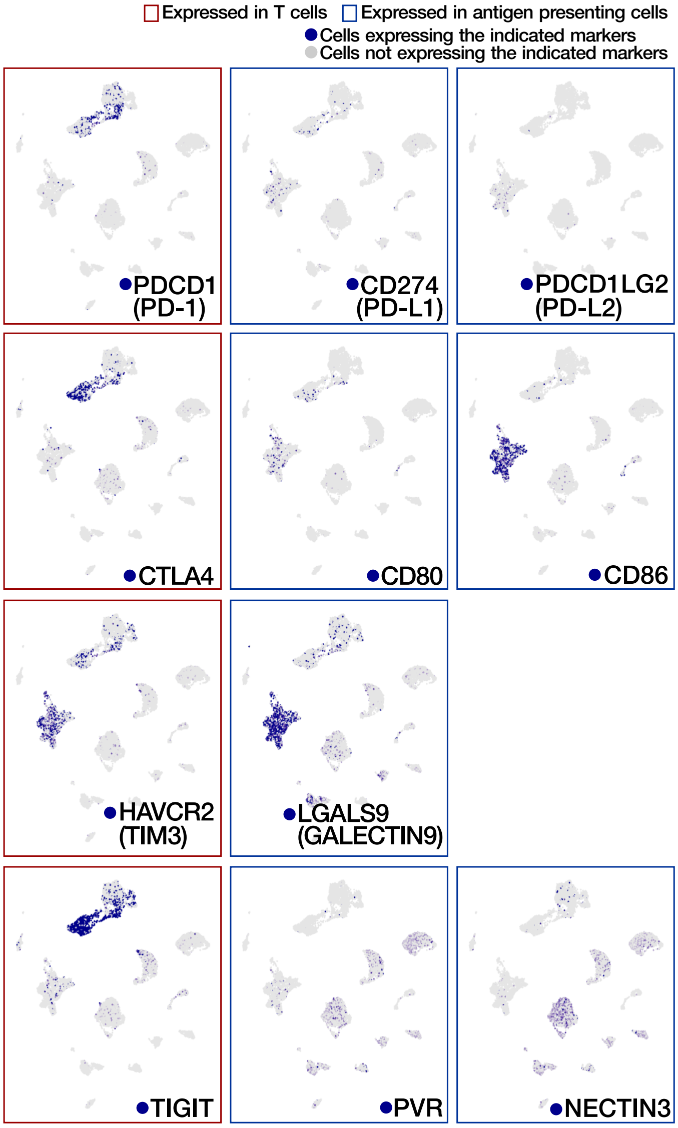

Supplementary Figure 11. Gene expression pattern of complementary co-inhibitory immune checkpoint molecules. Blue color indicates marker expressing cells.

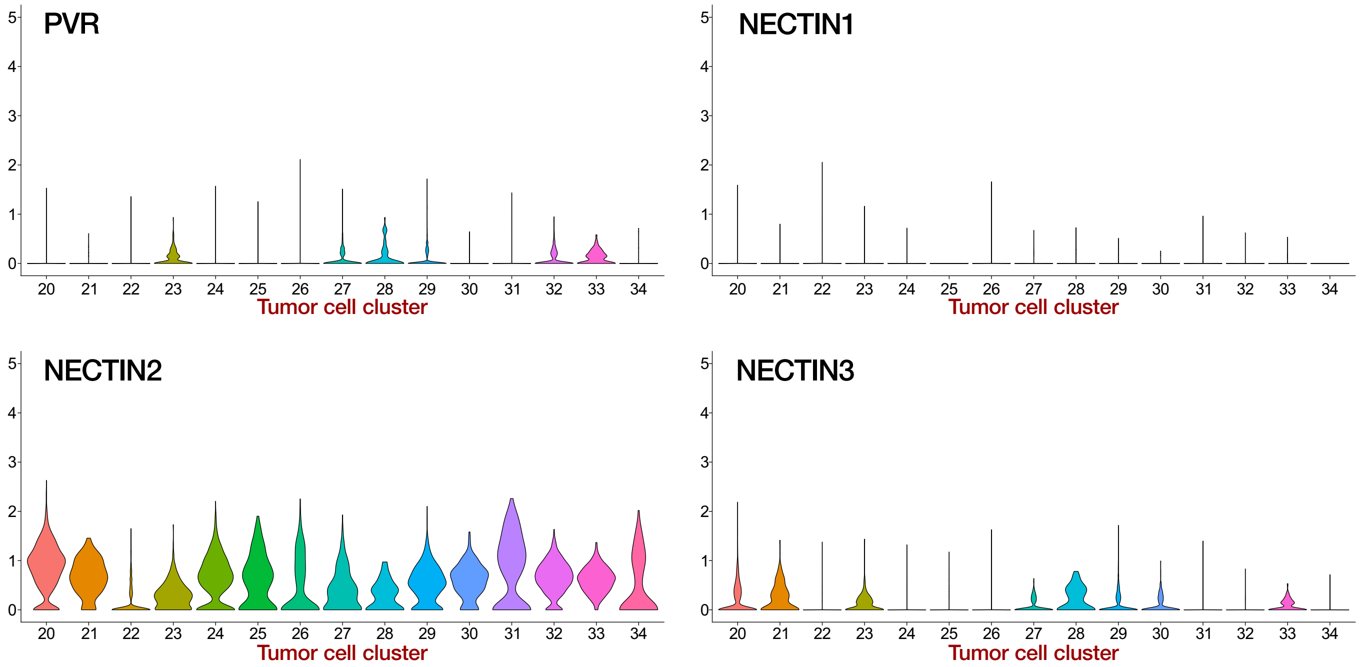

Supplementary Figure 12. NECTIN2 is the most prominently expressed in PVR family that interacts with TIGIT.

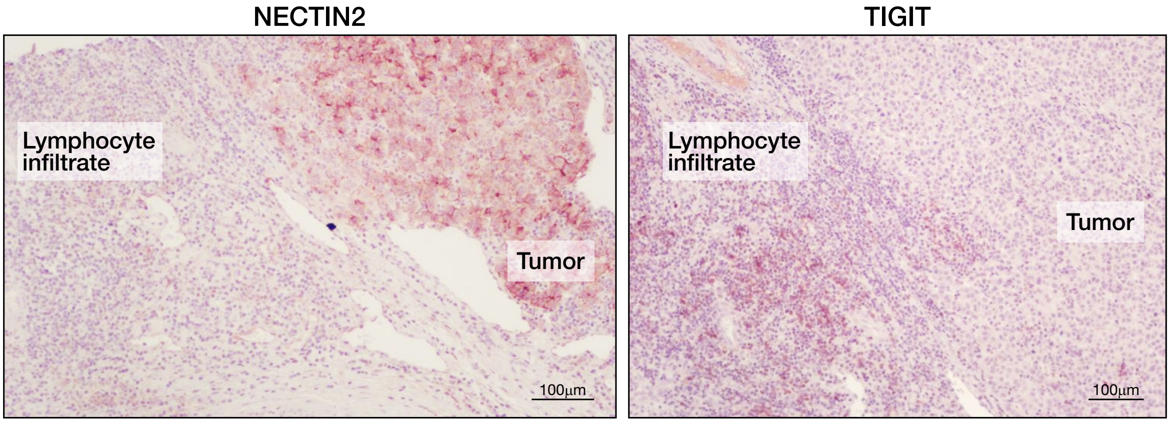

Supplementary figure 13. IHC staining of TIGIT and NECTIN2.
A representative case of HCC showing strong IHC positive staining for NECTIN2 in the tumor cells (upper right of the left panel) and positive staining for TIGIT in the surrounding lymphocytes (lower left of the right panel). Scale bar = 100μm.
More than 10 fields each under x40 and x100 magnification were examined.

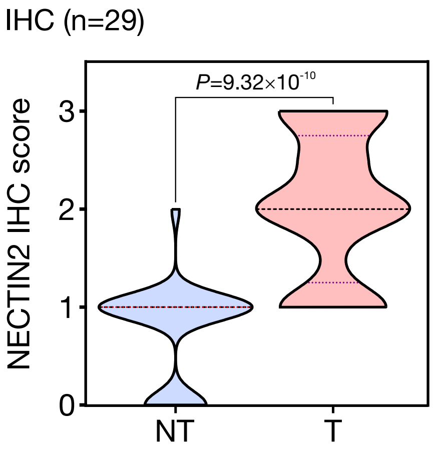

Supplementary Figure 14. Comparison of IHC of NECTIN2 on HCCs and the corresponding non-tumorous livers. There was significant upregulation of NECTIN2 in HCCs, as compared to non-tumorous livers. Source data are provided as a Source Data file.

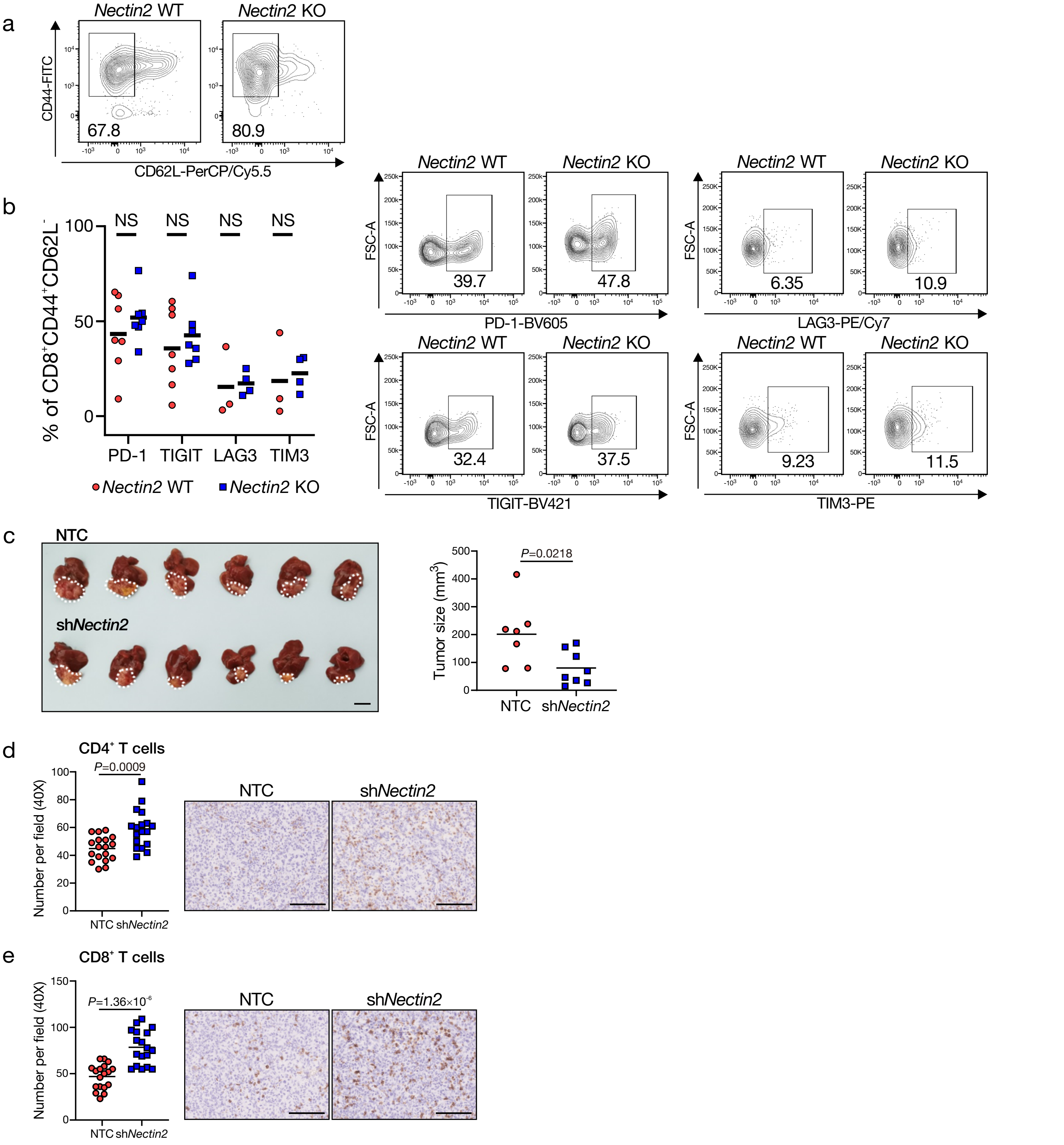

Supplementary Figure 15. Effect of *Nectin2* KO in HCC.
(a) Contour plots of the T effector cells in *Nectin2* WT and KO HCC tumors (Figure 5d).
(b) Expression of exhaustion markers (PD-1, TIGIT, LAG-3, TIM-3) in T effector cells. Experiment was performed with variable number of biologically independent samples (n number) (*Nectin2* WT: PD-1=7, TIGIT=7, LAG3=3, TIM3=3; *Nectin2* KO: PD-1=7, TIGIT=7, LAG3=4, TIM3=4)
(c) Representative picture and volume of tumors derived from Hepa1-6-NTC, -sh*Nectin2* cells. Scale bar = 1cm.
Experiment was performed with variable number of biologically independent samples (n number) (NTC=6, sh*Nectin2*=8)
(d-e) Representative pictures and quantification of CD4+ T cells and CD8+ T cells in HCC tumors by IHC staining. Scale bar = 100μm.
Experiment was performed with n=18 biologically independent samples per group.
(b-e) Student's t test. NS, not significant.
Source data are provided as a Source Data file.

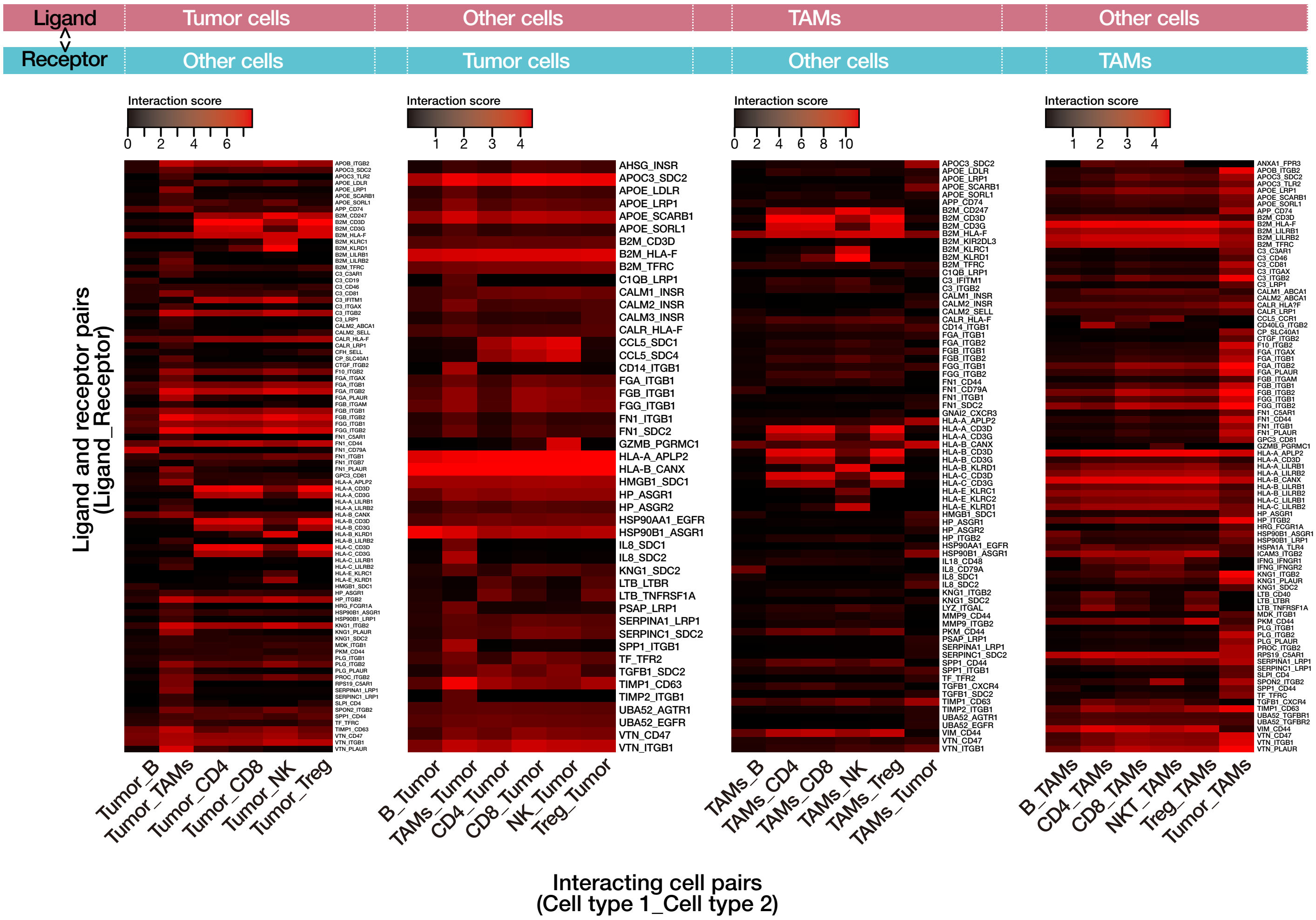

Supplementary Figure 16. Cell-cell communication between immune cells and APCs (TAMs and tumor cells) via ligand-receptor interactions. Extent of interaction was quantified through the calculation of interaction score, under different scenario (1: tumor cells contributing ligands; 2: tumor cells contributing receptors; 3: TAMs contributing ligands; 4: TAMs contributing receptors). In general, HCC tumor cells frequently interacted with immune cells.

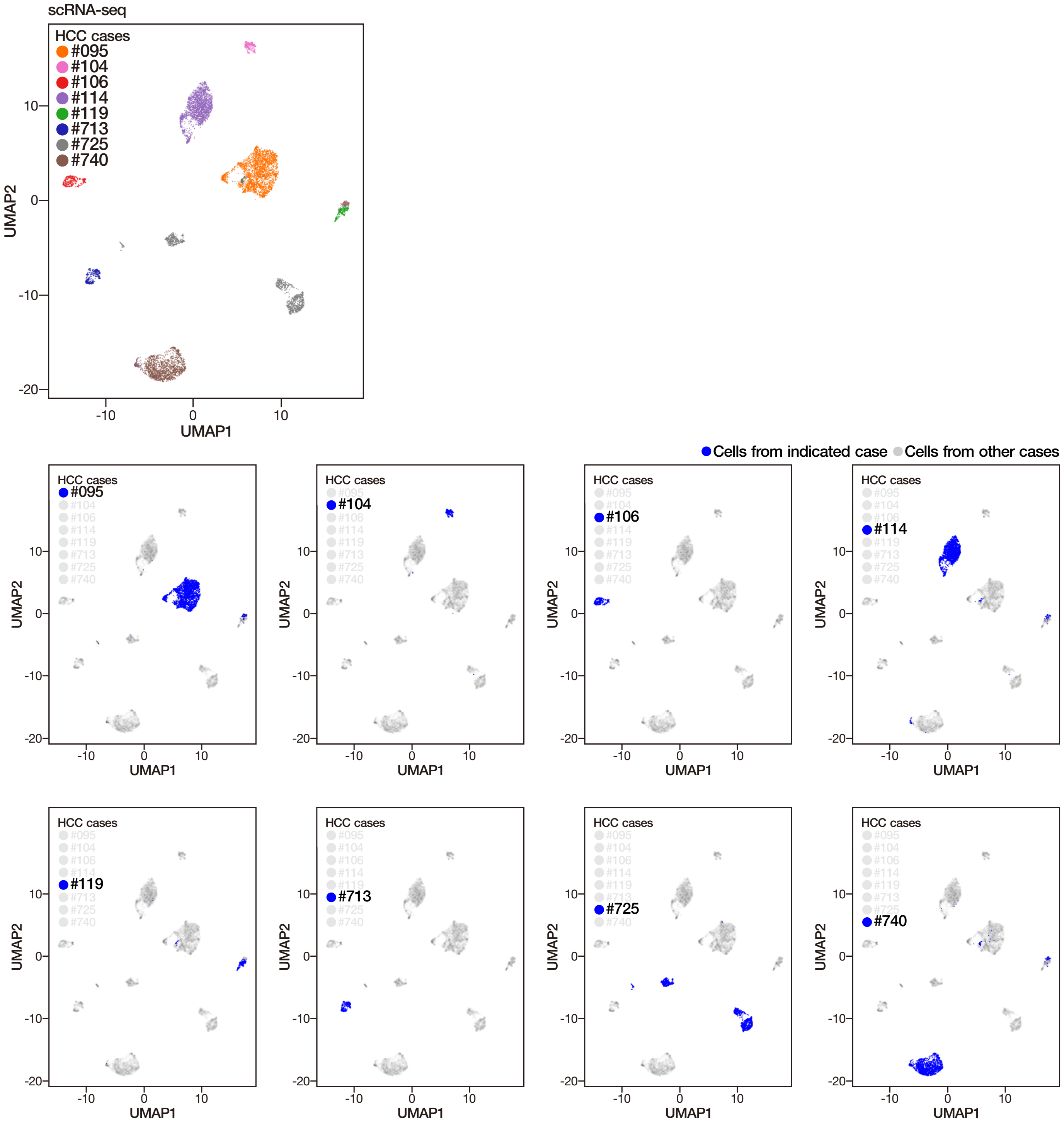

Supplementary Figure 17. HCC tumor cells stratified according to global transcriptomic profile.
Tumor cells mainly separated according to case identity into distinctive and tight cell clusters. There were also minority of tumor cells from individual cases that were not located in the major cell clusters of the cases. Therefore, each HCC case had the corresponding major and minor tumor cell populations. This is an evidence suggesting inter-tumoral heterogeneity is more prominent than the intra-tumoral one.

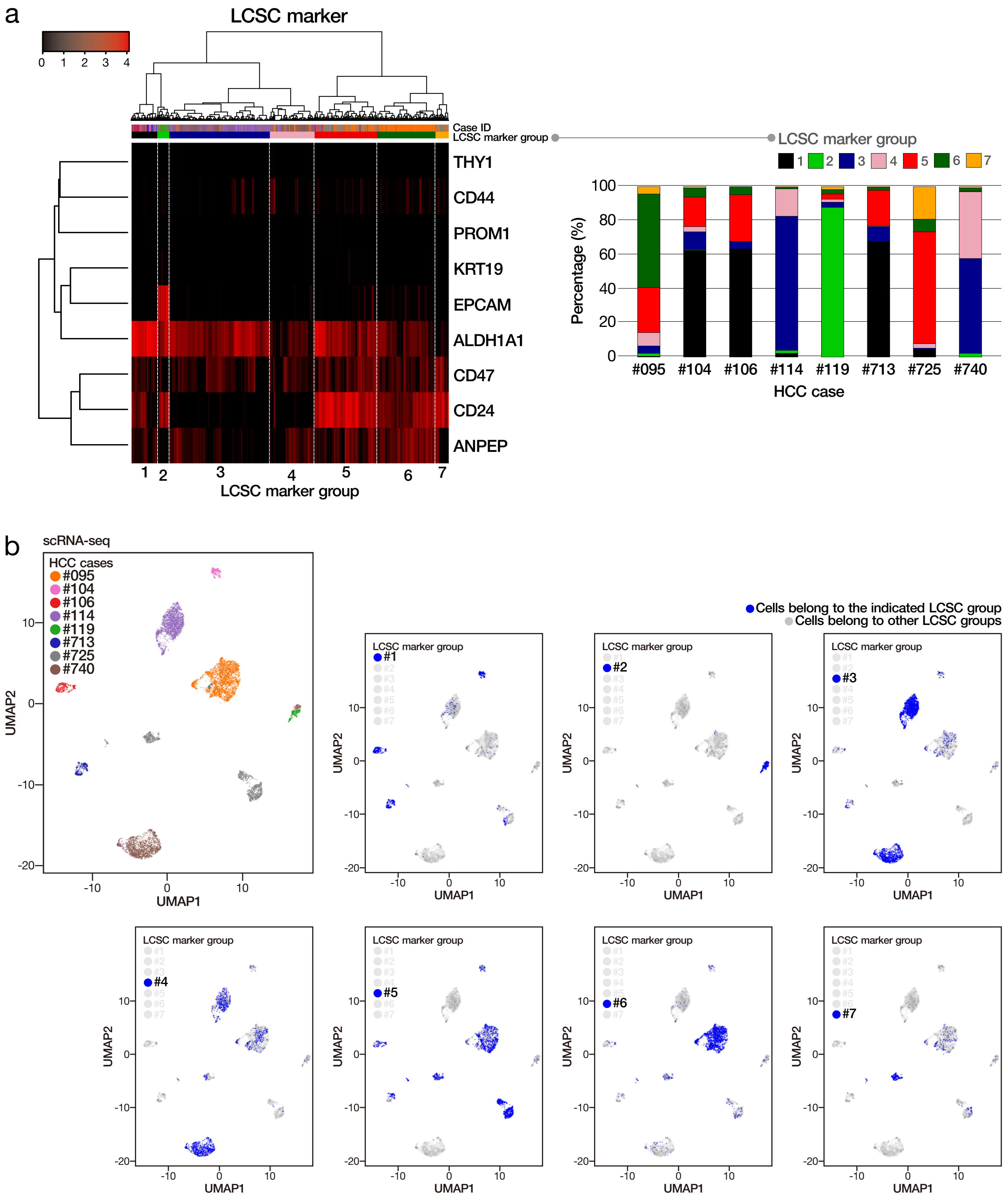

Supplementary Figure 18. LCSC marker expression status of HCC tumor cells.
(a) HCC tumor cells were classified into LCSC marker groups according to the unsupervised hierarchical clustering of gene expression of LCSC marker panel. Each HCC case was enriched with a major population of tumor cells of a certain LCSC marker group.
(b) Distribution of LCSC marker groups by correlating with case identity.

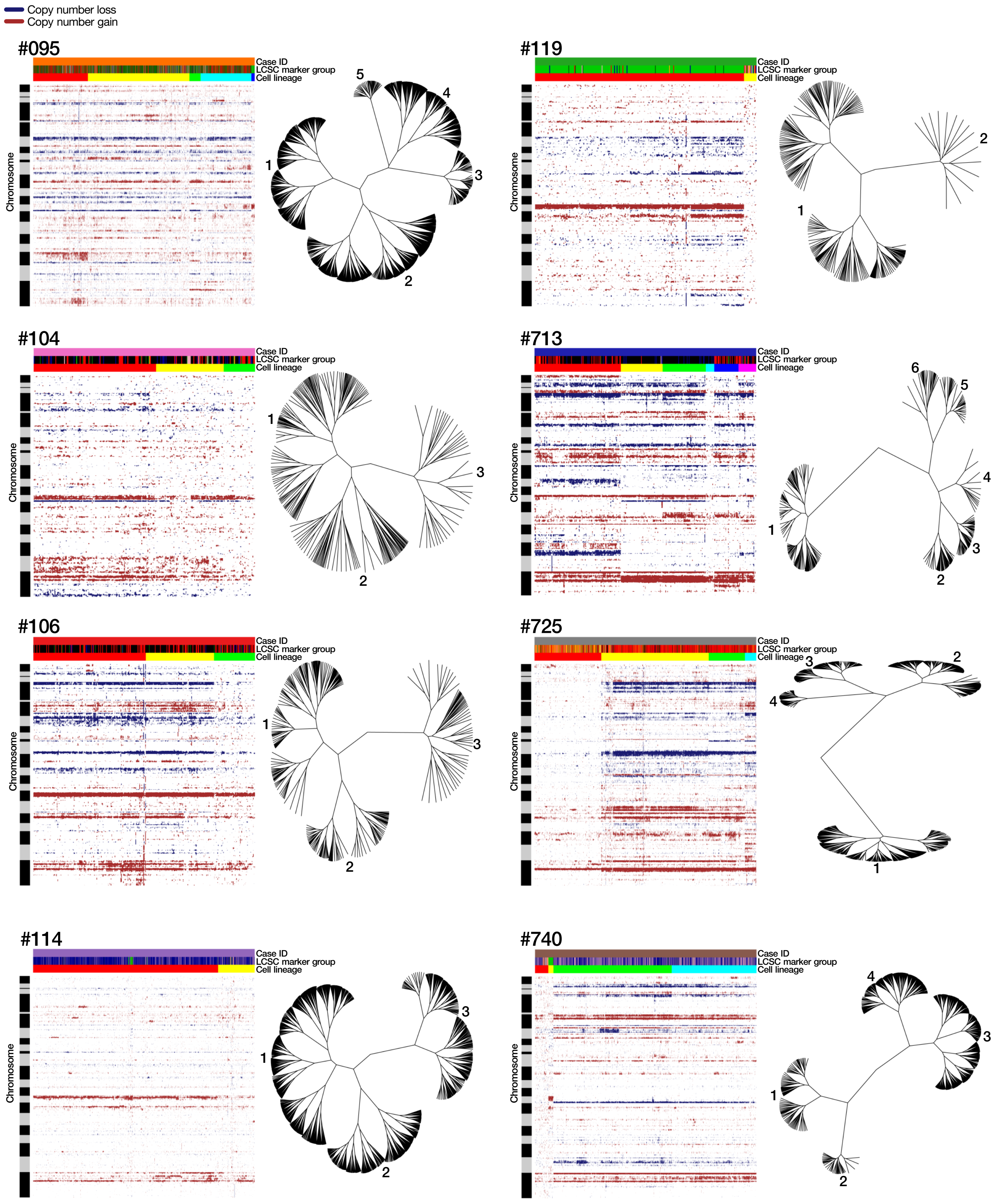

Supplementary Figure 19. CNV profile and the derived lineage hierarchy of tumor cells.
Lineage hierarchy of tumor cells were identified based on CNV profiles.

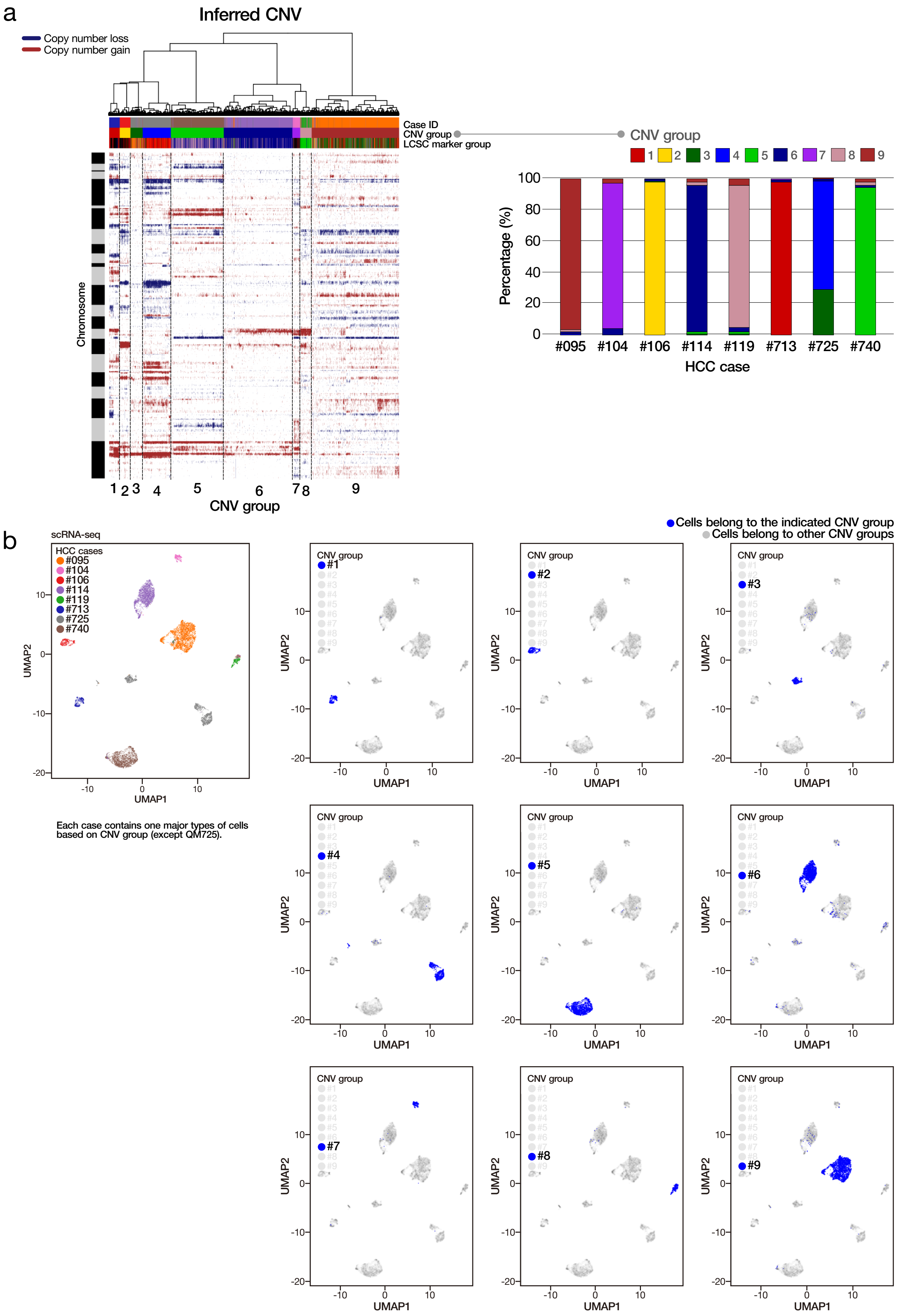

Supplementary Figure 20. CNV status of HCC tumor cells.
(a) HCC tumor cells were classified into CNV groups according to the unsupervised hierarchical clustering of CNV profile. Each HCC case was enriched with a major population of tumor cells of a certain CNV group.
(b) Distribution of CNV groups by correlating with case identity.

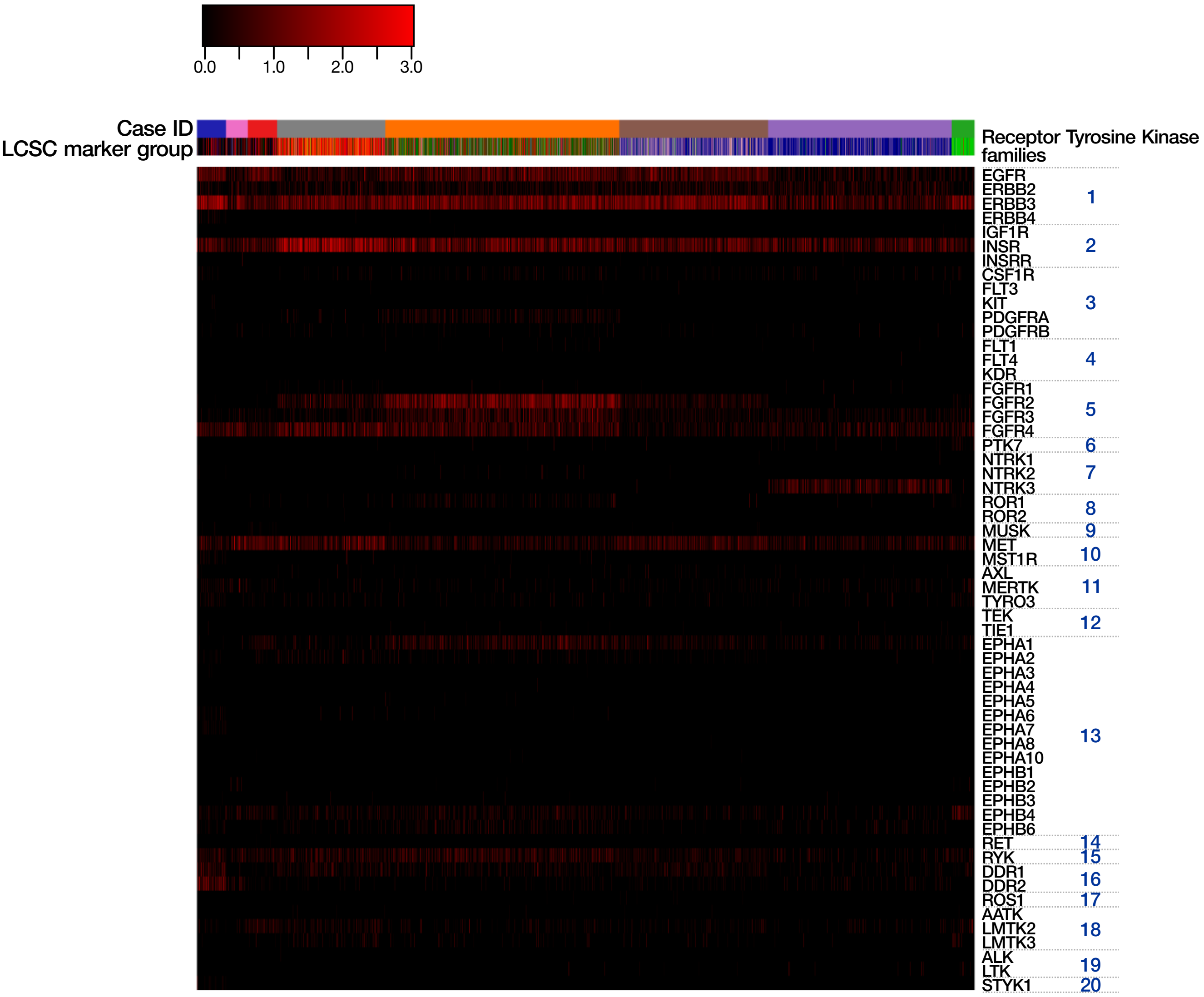

Supplementary Figure 21. Gene expression of RTK families in HCC tumor cells.
Expression of RTK genes was enriched in case-specific manner.

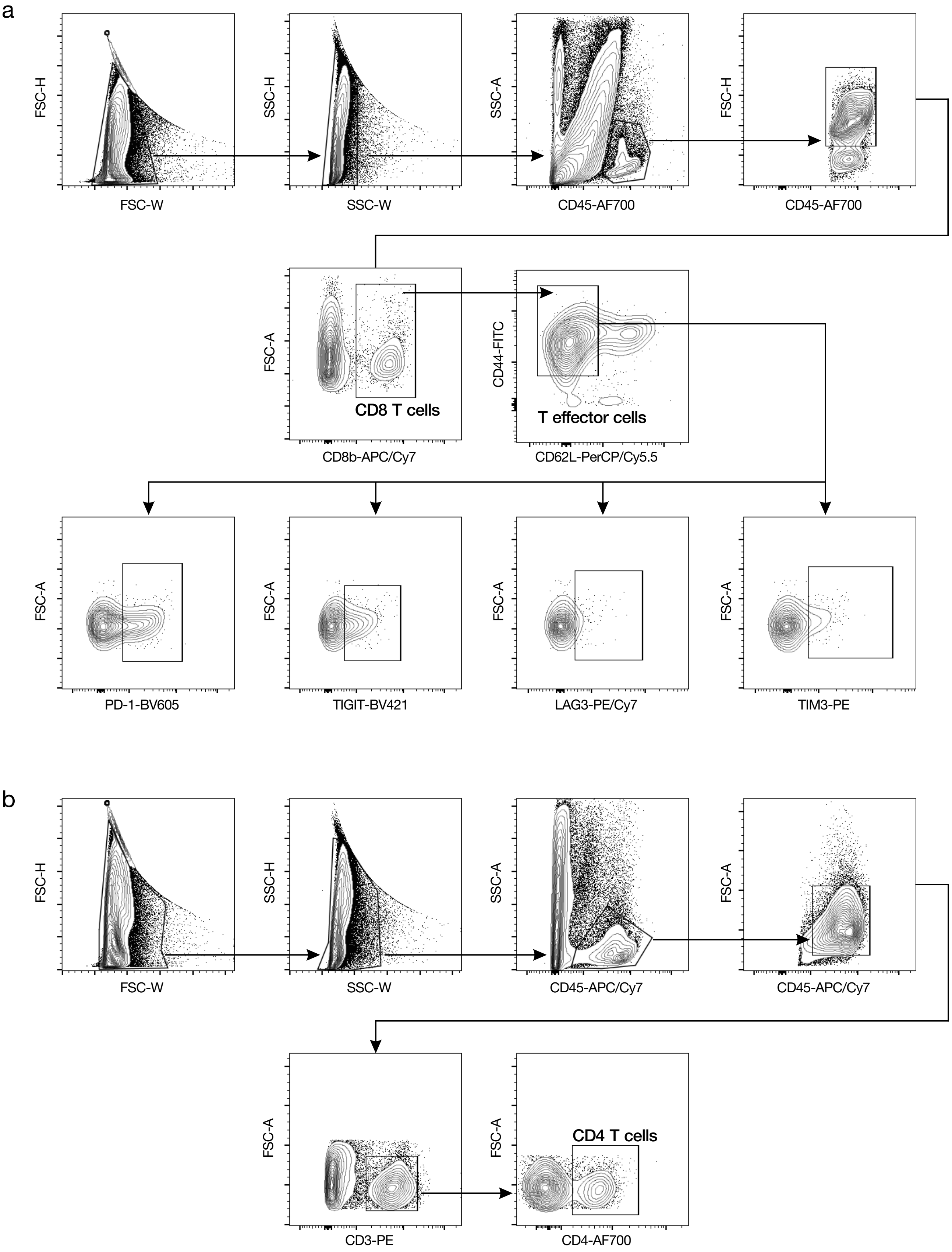

Supplementary Figure 22. Gating strategies for tumor-infiltrating lymphocytes analysis in Figure 5 and Supplementary Figure 15.
(a) Gating strategy for CD8 T cells, T effector cells and their exhaustion markers.
(b) Gating strategy for CD4 T cells.

Supplementary Table 1. Demographic and pathological data of the HCC patients.

**Case number**	**#713**	**#725**	**#740**	**#095**	**#104**	**#106**	**#114**	**#119**
Gender	M	M	M	F	M	M	M	M
Age (years)	64	61	54	73	71	68	61	45
No. of tumor nodules	1	2	1	1	1	1	1	1
Tumor size (cm)	8.5	2	3.6	9	4.5	4.9	5	13
Tumor encapsulation	Absent	Absent	Present	Absent	Present	Present	Absent	Absent
Cellular differentiation (by Edmondson grading)	III-IV	III-IV	III-IV	III-IV	I-II	III-IV	III-IV	III-IV
Venous invasion	Present	Present	Present	Present	Present	Absent	Present	Present
Tumor microsatellite formation	Present	Present	Present	Present	Present	Absent	Present	Present
Liver invasion	Present	Present	Absent	Present	Absent	Absent	Present	Present
Background liver	Cirrhosis	Cirrhosis	Cirrhosis	CH	NS	CH	Cirrhosis	CH
HBV status	Positive	Positive	Positive	Positive	Positive	Positive	Positive	Positive
HCV status	Negative	Negative	Negative	Negative	Negative	Negative	Negative	Negative
HDV status	NA	NA	NA	NA	NA	NA	NA	NA
HBeAg	Negative	Negative	Negative	NA	NA	NA	NA	NA
HBV DNA (IU/ml)	<10	<10	1.7e3	<10	<10	62	1.9e6	2.4e5
Hyperlipidemia	Yes	No	No	Yes	No	No	No	No
Diabetes	Yes	No	No	Yes	No	No	No	No
Alcoholism	No	No	No	No	Yes	No	No	No

M, male; F, female; CH, chronic hepatitis; NS, non-specific changes; NA, not available.

Supplementary Table 2. Statistics of the scRNA-seq dataset.

**Case number**	**#713**	**#725**	**#740**	**#095**	**#104**	**#106**	**#114**	**#119**
Number of reads (millions)	293.9	312.9	1076.7	1134.3	286.9	290.4	1055.8	1008.6
Estimated number of cells	1672	2770	8720	8296	533	760	12162	8732
Mean reads per cell	175786	112952	123475	136739	538246	382099	86815	115505
Valid barcodes	97.8%	97.6%	97.7%	97.7%	93.7%	97.4%	97.9%	97.9%
Sequencing saturation	84.5%	77.0%	75.5%	82.1%	94.9%	84.0%	85.8%	93.4%
Q30 bases in barcode	95.0%	92.8%	97.1%	97.2%	88.3%	95.1%	97.2%	97.3%
Q30 bases in RNA read	80.8%	69.1%	93.3%	93.7%	68.1%	76.4%	92.8%	93.3%
Q30 bases in UMI	95.7%	93.9%	97.2%	97.3%	93.9%	95.9%	97.3%	97.3%
Total genes detected	21601	22607	23697	25043	17562	20415	23496	21932
Number of cells after QC	844	1796	2606	4779	311	510	5177	2031

Supplementary Table 3. Gene markers used for cell type identification.

**Category**	**Gene marker**
Cell type markers	
Pan-leukocyte	PTPRC
Monocyte	CD14
Pan-macrophage	CD68
M2 macrophage	CD163
Dendritic cell	ITGAX
Myeloid-derived suppressor cell	ITGAM<br>CD33
B cell	CD19<br>CD79A
Natural killer cell	NCAM1<br>FCGR3A
Pan-T cell	CD3E
CD4 T	CD4<br>IL7R
CD8 T	CD8A<br>NKG7
Regulatory T	CD4<br>IL2RA<br>FOXP3
Endothelial cell	PECAM1<br>CD34
HCC tumor markers	AFP<br>GPC3
LCSC markers	ALDH1A1<br>EPCAM<br>KRT19<br>ANPEP<br>CD24<br>CD44<br>CD47<br>THY1<br>PROM1

Supplementary Table 4. Cell type composition in different HCC cases.

**Cell type**	**#713**	**#725**	**#740**	**#095**	**#104**	**#106**	**#114**	**#119**
B cells	146	100	7	46	0	21	58	65
Macrophages	354	383	381	938	26	44	704	232
T cells	5	51	479	1049	20	104	2268	1466
Tumor cells	339	1262	1735	2731	255	341	2142	266
Other cells	0	0	4	15	10	0	5	2

Supplementary Table 5. Correlation of the LAIR1 and CD163 expression by IHC in human HCC (n=29). Chi-square test (2-sided).

		**CD163 expression**		
		**High**	**Low**	**Chi-square *P***
**LAIR1 expression**	**High**	9	2	
	**Low**	2	16	0.0001

Supplementary Table 6. Correlation of the TIGIT and NECTIN2 expression by IHC in human HCC (n=29). Chi-square test (2-sided).

		**NECTIN2 expression**		
		**High**	**Low**	**Chi-square *P***
**TIGIT expression**	**High**	8	3	
	**Low**	6	12	0.039

Supplementary Table 7. Correlation of TIGIT and NECTIN2 expression by IHC in human non-HCC, HBV-associated cirrhotic livers (n=22). Chi-square test (2-sided).

		**NECTIN2 expression**		
		**High**	**Low**	**Chi-square *P***
**TIGIT expression**	**High**	2	1	
	**Low**	9	10	0.534

Supplementary Table 8. Distribution of LCSC marker groups in tumor cells of different HCC cases.

**LCSC marker group**	**#713**	**#725**	**#740**	**#095**	**#104**	**#106**	**#114**	**#119**
Gp1	231	58	2	14	160	217	51	0
Gp2	0	0	36	44	1	0	33	234
Gp3	29	11	965	127	27	15	1693	8
Gp4	1	33	685	208	8	0	338	5
Gp5	72	828	7	729	44	94	7	8
Gp6	5	92	30	1495	13	15	14	7
Gp7	1	240	10	114	2	0	6	4

Supplementary Table 9. Distribution of CNV groups in tumor cells of different HCC cases.

**CNV group**	**#713**	**#725**	**#740**	**#095**	**#104**	**#106**	**#114**	**#119**
Gp1	331	0	0	0	0	0	1	0
Gp2	0	0	0	0	0	334	0	0
Gp3	0	365	0	2	0	1	13	2
Gp4	0	875	0	1	0	3	1	0
Gp5	1	0	1637	2	0	0	17	2
Gp6	5	17	30	52	10	3	2021	8
Gp7	2	0	0	0	238	0	5	0
Gp8	0	0	35	36	0	0	43	243
Gp9	0	5	33	2638	7	0	41	11

Supplementary Table 10. Information of the sgRNA and shRNA sequences.

**sgRNA/shRNA clones**	**Target nucleotide sequences**
Nectin2-KO1 sgRNA	CACCGCGGGTACGAGTGCTTCCCG
Nectin2-KO2 sgRNA	CACCGTCTGGACGGCGGGAGGACTG
Nectin2-KO3 sgRNA	CACCGCGGGTACGAGTGCTTCCCG
Nectin2-KO (HDTVi) sgRNA	CACCGAGCGGCAACAACGGCAGCGT
Nectin2-KD shRNA	GAAGGACCTCCCTCCTATAAA
LAIR1-KD shRNA	GACCTGGCTGTTGATGTTCTA

Supplementary Table 11. Information of primary antibodies for flow cytometry, IHC staining and Nectin2 neutralizing antibody.

Primary antibodies for flow cytometry

Antibody	Fluorochrome	Supplier	Clone	Dilution
CD45	AF700	Biolegend	30-F11	1:100
CD8b	APC/Cy7	Biolegend	YTS156.7.7	1:100
CD44	FITC	Biolegend	IM7	1:100
CD62L	PerCP/Cy5.5	Biolegend	MEL-14	1:100
PD-1	BV605	Biolegend	29F.1A12	1:50
TIGIT	BV421	Biolegend	1G9	1:100
LAG-3	PE/Cy7	Biolegend	C9B7W	1:100
TIM-3	PE	Biolegend	RM3-23	1:100
CD45	APC/Cy7	Biolegend	30-F11	1:100
CD3	PE	Biolegend	17A2	1:100
CD4	AF700	Biolegend	GK1.5	1:100
CD25	AF488	eBioscience	eBio7D4	1:100
FoxP3	PerCP/Cy5.5	eBioscience	FJK-16s	1:100

Primary antibodies for IHC staining

Antibody	Supplier	Clone	Dilution
CD4	Cell Signaling Technology	D7D2Z	1:100
CD8a	Cell Signaling Technology	D4W2Z	1:200

Antibody for T cell proliferation assay

Antibody	Supplier	Clone
Nectin2	R&D Systems	829038
