# Supplementary material for: Single-cell RNA sequencing shows the immunosuppressive landscape and tumor heterogeneity of HBV-associated hepatocellular carcinoma
Source: Nat Commun. 2021 Jun 17;12:3684. doi: 10.1038/s41467-021-24010-1 (PMC8211687; doi:10.1038/s41467-021-24010-1)
Supplement: Supplementary file 3 — Reporting Summary [file 41467_2021_24010_MOESM3_ESM.pdf]

nature research

Corresponding author(s): DWH Ho, IOL Ng

Last updated by author(s): May 6, 2021

Reporting Summary

Nature Research wishes to improve the reproducibility of the work that we publish. This form provides structure for consistency and transparency in reporting. For further information on Nature Research policies, see our Editorial Policies and the Editorial Policy Checklist.

Statistics

For all statistical analyses, confirm that the following items are present in the figure legend, table legend, main text, or Methods section.

n/a	Confirmed	
☐	☒	The exact sample size (*n*) for each experimental group/condition, given as a discrete number and unit of measurement
☐	☒	A statement on whether measurements were taken from distinct samples or whether the same sample was measured repeatedly
☐	☒	The statistical test(s) used AND whether they are one- or two-sided<br>*Only common tests should be described solely by name; describe more complex techniques in the Methods section.*
☒	☐	A description of all covariates tested
☐	☒	A description of any assumptions or corrections, such as tests of normality and adjustment for multiple comparisons
☐	☒	A full description of the statistical parameters including central tendency (e.g. means) or other basic estimates (e.g. regression coefficient) AND variation (e.g. standard deviation) or associated estimates of uncertainty (e.g. confidence intervals)
☐	☒	For null hypothesis testing, the test statistic (e.g. *F*, *t*, *r*) with confidence intervals, effect sizes, degrees of freedom and *P* value noted<br>*Give P values as exact values whenever suitable.*
☒	☐	For Bayesian analysis, information on the choice of priors and Markov chain Monte Carlo settings
☐	☒	For hierarchical and complex designs, identification of the appropriate level for tests and full reporting of outcomes
☐	☒	Estimates of effect sizes (e.g. Cohen's *d*, Pearson's *r*), indicating how they were calculated

Our web collection on statistics for biologists contains articles on many of the points above.

Software and code

Policy information about availability of computer code

Data collection	We used the Cell Ranger (version 3.0) from 10X Genomics to perform initial data demultiplexing, read alignment, UMI counting and annotation on the raw read data.
Data analysis	We used the kBET algorithm (v0.99.5) for batch effect checking, Seurat package (v2.3) for quality control filtering, data preprocessing, scaling, normalization, Louvain clustering and data visualization, UMAP algorithm for nonlinear dimensionality reduction, the method by Tirosh et al. Nature 2016 (doi: 10.1038/nature20123) for inferring CNV status, Monocle (v2) for pseudo-time cell trajectory analysis, the method by Kumar et al. Cell Reports 2018 (doi: 10.1016/j.celrep.2018.10.047) for cell-cell interaction analysis and GraphPad Prism 6 for data plotting. FlowJo was used for analyzing flow cytometry data. Default parameters were used unless otherwise specified. Details are described in the Materials and Methods.

For manuscripts utilizing custom algorithms or software that are central to the research but not yet described in published literature, software must be made available to editors and reviewers. We strongly encourage code deposition in a community repository (e.g. GitHub). See the Nature Research guidelines for submitting code & software for further information.

Data

Policy information about availability of data

All manuscripts must include a data availability statement. This statement should provide the following information, where applicable:

- Accession codes, unique identifiers, or web links for publicly available datasets
- A list of figures that have associated raw data
- A description of any restrictions on data availability

The sequence data that support the findings of this study have been deposited in NCBI Sequence Read Archive with the accession code SRP318499.

Field-specific reporting

Please select the one below that is the best fit for your research. If you are not sure, read the appropriate sections before making your selection.

☒ Life sciences ☐ Behavioural & social sciences ☐ Ecological, evolutionary & environmental sciences

For a reference copy of the document with all sections, see nature.com/documents/nr-reporting-summary-flat.pdf

Life sciences study design

All studies must disclose on these points even when the disclosure is negative.

Sample size	Sequencing depth and number of libraries were defined to allow support for the paper's main conclusions. We estimated the required number of single cells to be sequenced based on the Navin. Genome Research (2015) (Pubmed ID: 26430160). For mouse experiment, the required sample size was estimated based on our similar study performed previously (Ho et al. Cancer Letters 2019; Pubmed ID: 31195060).
Data exclusions	Except for data filtering, no data were excluded from the analysis.
Replication	Findings derived from single-cell RNA sequencing experiment were confirmed using independent in-house and TCGA bulk-cell RNA-seq datasets, immunohistochemistry, immunofluorescence staining and in vivo mouse models. For cell and mouse experiments, at least 3 biological replicates were performed to verify the reproducibility. All replication attempts were successful.
Randomization	Patients were randomly selected from the available sample cohort. For mouse experiments, mice were randomly allocated into either treatment or control group. For cell experiments, cells were randomly assigned to either treatment or control group.
Blinding	Blinding was not applicable in this study. For sRNA-seq data analysis, due to no treatment was done, there was no need for blinding procedure. For experiments, blinding was not possible because individual groups of cells or mice received different treatments. Treatment, data recording and analysis were done by the same investigators throughout the study.

Reporting for specific materials, systems and methods

We require information from authors about some types of materials, experimental systems and methods used in many studies. Here, indicate whether each material, system or method listed is relevant to your study. If you are not sure if a list item applies to your research, read the appropriate section before selecting a response.

Materials & experimental systems

n/a	Involved in the study	
☐	☒	Antibodies
☐	☒	Eukaryotic cell lines
☒	☐	Palaeontology and archaeology
☐	☒	Animals and other organisms
☐	☒	Human research participants
☒	☐	Clinical data
☒	☐	Dual use research of concern

Methods

n/a	Involved in the study	
☒	☐	ChIP-seq
☐	☒	Flow cytometry
☒	☐	MRI-based neuroimaging

Antibodies

Antibodies used	IHC was performed on formalin-fixed, paraffin-embedded (FFPE) sections, using anti-NECTIN2 (Sigma-Aldrich, HPA012759, 1:300), anti-TIGIT (Abcam, Ab243903, 1:200), anti-CD163 (Abcam, Ab189915, 1:500) and anti-LAIR1 (Sigma-Aldrich, HPA011155, 1:500) rabbit antibodies. Information of the other antibodies used are listed in Supplementary Table 11.
Validation	All antibodies used are commercially available and their manufacturers provided their validation documents. They were validated for flow cytometry, Western blot, IF and/or IHC staining.<br>https://www.sigmaaldrich.com/catalog/product/sigma/hpa012759<br>https://www.abcam.com/tigit-antibody-blr047f-bsa-free-ab243903.html<br>https://www.abcam.com/cd163-antibody-epr14643-36-c-terminal-ab189915.html<br>https://www.sigmaaldrich.com/catalog/product/sigma/hpa011155<br>https://www.biolegend.com/en-us/products/alexa-fluor-700-anti-mouse-cd45-antibody-3407<br>https://www.biolegend.com/en-us/products/apc-cyanine7-anti-mouse-cd8b-antibody-10021<br>https://www.biolegend.com/en-us/products/fitc-anti-mouse-human-cd44-antibody-314<br>https://www.biolegend.com/en-us/products/percp-cyanine5-5-anti-mouse-cd62l-antibody-4272<br>https://www.biolegend.com/en-us/products/brilliant-violet-605-anti-mouse-cd279-pd-1-antibody-7648<br>https://www.biolegend.com/en-us/products/brilliant-violet-421-anti-mouse-tigit-vstm3-antibody-14595<br>https://www.biolegend.com/en-us/products/pe-cyanine7-anti-mouse-cd223-lag-3-antibody-14782

https://www.biolegend.com/en-us/products/pe-cyanine7-anti-mouse-cd366-tim-3-antibody-12948
https://www.biolegend.com/en-us/products/apc-cyanine7-anti-mouse-cd45-antibody-2530
https://www.biolegend.com/en-us/search-results/pe-anti-mouse-cd3-antibody-47
https://www.biolegend.com/en-us/products/alexa-fluor-700-anti-mouse-cd4-antibody-3385
https://www.thermofisher.com/antibody/product/CD25-Antibody-clone-eBio7D4-7D4-Monoclonal/53-0252-82
https://www.thermofisher.com/antibody/product/FOXP3-Antibody-clone-FJK-16s-Monoclonal/45-5773-82
https://www.cellsignal.com/products/primary-antibodies/cd4-d7d2z-rabbit-mab/25229
https://www.cellsignal.com/products/primary-antibodies/cd8a-d4w2z-xp-rabbit-mab-mouse-specific/98941
https://www.rndsystems.com/products/mouse-nectin-2-cd112-antibody-829038_mab3869

Eukaryotic cell lines

Policy information about cell lines

Cell line source(s)	Hepa1-6 cells: American Type Culture Collection.
Authentication	Passed quality controls for characterization (by genotyping), viability and sterility by the manufacturer.
Mycoplasma contamination	Passed mycoplasma test by the manufacturer.
Commonly misidentified lines (See ICLAC register)	No commonly misidentified cell lines were used in the study.

Animals and other organisms

Policy information about studies involving animals; ARRIVE guidelines recommended for reporting animal research

Laboratory animals	5-to-7-week old C57/BL6N male mice. Mice were housed with a dark/light cycle of 12 hour, ambient temperature of 22oC and humidity of 30-70%.
Wild animals	No wild animals were used in the study.
Field-collected samples	No field collected samples were used in the study.
Ethics oversight	All animal studies were approved by the Committee on the Use of Live Animals in Teaching and Research, the University of Hong Kong, and performed under the Animals (Control of Experiments) Ordinance of Hong Kong.

Note that full information on the approval of the study protocol must also be provided in the manuscript.

Human research participants

Policy information about studies involving human research participants

Population characteristics	The demographic and pathological data of the eight randomly selected HCC cases are shown in Supplementary Table 1.
Recruitment	Eight HBV-associated HCC cases surgically resected from patients were randomly selected with the following criteria: (1) HBV-associated, and (2) more than 1000 viable single cells obtained in each case. HBV-associated HCCs were selected to have a uniform etiological background of HCC, because >80% of our HCC cases locally are associated with chronic HBV infection. The HCC tumor tissues were obtained immediately in the operation theater after surgical resection at Queen Mary and Queen Elizabeth Hospitals of Hong Kong. We only examined HBV-associated HCCs in the current study and our findings should not be extrapolated to HCC of other etiologies. Except this, there are no other potential bias for recruitment in our study.
Ethics oversight	The study was approved by the Institutional Review Board of the University of Hong Kong/Hospital Authority Hong Kong West Cluster (UW 17-056) and informed consents were obtained from patients.

Note that full information on the approval of the study protocol must also be provided in the manuscript.

Flow Cytometry

Plots

Confirm that:

- [x] The axis labels state the marker and fluorochrome used (e.g. CD4-FITC).
- [x] The axis scales are clearly visible. Include numbers along axes only for bottom left plot of group (a 'group' is an analysis of identical markers).
- [x] All plots are contour plots with outliers or pseudocolor plots.
- [x] A numerical value for number of cells or percentage (with statistics) is provided.

Methodology

Sample preparation	Single cell suspensions from mouse tumors were prepared prior to flow cytometry analysis. Tumors harvested from HCC-bearing mice were cut into fine pieces and were suspended in DMEM/F12 medium. 30µL of Liberase (2.5mg/mL; Sigma Aldrich, St Louis, USA) and 60µL of DNase I (10mg/mL; Sigma Aldrich) were added. Tissues were dissociated with GentleMACS Dissociator (Miltenyi Biotech, Germany). Red blood cells were lysed with ACK lysing buffer. The filtered cell suspensions were washed and reconstituted in cell staining buffer for flow cytometry.
Instrument	BD LSRFortessa Flow Cytometer and NovoCyte Advanteon BVR.
Software	Data were analyzed by FlowJo (Tree Star Inc, Ashland, OR).
Cell population abundance	Cell sorting is not involved in these two animal studies.
Gating strategy	Details are described in the Methods section and illustrated in Supplementary Figure. Briefly, Live cells are gated by FSC and SSC. Positive and negative cell populations are defined based on the respective isotype controls.

☒ Tick this box to confirm that a figure exemplifying the gating strategy is provided in the Supplementary Information.
